# Self-assembled structures as a material basis for indirect pharmacology in Traditional Chinese Medicine

**DOI:** 10.1186/s13020-026-01471-4

**Published:** 2026-07-30

**Authors:** Jian Li, Wei Zhang, Jiayu Ye, Hualin Peng

**Affiliations:** 1 Fujian Key Laboratory of Traditional Chinese Veterinary Medicine and Animal Health, College of Animal Science, Fujian Agriculture and Forestry University, 15 Shangxiadian Road, Fuzhou, 350002 Fujian People’s Republic of China; 2 University Key Laboratory for Integrated Chinese Traditional and Western Veterinary Medicine and Animal Healthcare in Fujian Province, Fuzhou, 350002 People’s Republic of China

**Keywords:** Indirect pharmacology, Supramolecular chemistry, Trans-organ communication, Gut-liver axis, Systems medicine, TCM decoctions

## Abstract

Indirect pharmacology is increasingly invoked to explain how Traditional Chinese Medicine (TCM) and natural products produce therapeutic effects through inter-organ communication and distributed physiological regulation rather than through direct action on a single molecular target. In this context, most mechanistic discussions emphasize downstream mediators, including microbial metabolites, immune signals, and endocrine factors, but pay less attention to the material form in which complex preparations are administered. This review examines the possibility that self-assembled structures in decoctions constitute an underappreciated upstream determinant of indirect pharmacology. Decoctions are not simply molecular solutions; during preparation and after exposure to biological environments, they can form colloids, aggregates, and multicomponent co-assemblies. These material states may influence intestinal localization, mucosal retention, microbial access, barrier interactions, and the spatial pattern of mediator generation. We synthesize evidence from two related lines of research: naturally occurring aggregates in traditional decoctions and bottom-up assemblies constructed from isolated phytochemicals. Across representative case studies, current evidence supports a role for supramolecular organization in shaping exposure and interface-level behavior, but direct causal links to defined mediator chains and distal organ outcomes remain limited. We therefore outline methodological strategies for testing structure-interface-mediator relationships more rigorously in future studies.

## Introduction

Indirect pharmacology is increasingly used to describe therapeutic effects that arise from inter-organ communication and network behavior rather than from modulation of a single target in a single organ [57]. This framework is particularly useful for understanding multi-organ disorders, such as metabolic disease [53], neurodegeneration [7], and fibrosis [23], because these conditions are sustained by disturbed crosstalk among the gut, liver, immune system, brain, and related tissues. Traditional Chinese Medicine (TCM) and natural products are often applied in these settings and are commonly described in terms of holistic regulation. The challenge for systems medicine is to translate “holistic regulation” into mechanistic statements that can be evaluated experimentally.

Indirect pharmacology usually centers on downstream mediators, such as endogenous metabolites, immune components, and microbiota-derived molecules, and on the anatomical and functional routes that connect organs, such as the gut–liver and gut–brain axes [3, 16]. This mediator-centered view is necessary, but it also leaves an upstream issue insufficiently examined: what physical forms of a complex TCM preparation actually enter the body, persist at interfaces, and trigger those mediator changes? In decoctions, the administered material is rarely a fully dispersed solution of purified molecules. Instead, it is a multicomponent system generated under heating, concentration, dilution, and changing chemical conditions that can favor physicochemical organization. Published studies have reported that decoctions can generate self-assembled structures (colloids, nano/micro-aggregates, and co-assemblies) during preparation and after encountering biological environments [61, 67]. Existing reviews have covered self-assembly mechanisms and biomedical applications in broad terms [13, 62]; here, we focus on a narrower point— how far current evidence supports a role for these material states in shaping mediator-linked, trans-organ pharmacology.

**Fig. 1:**
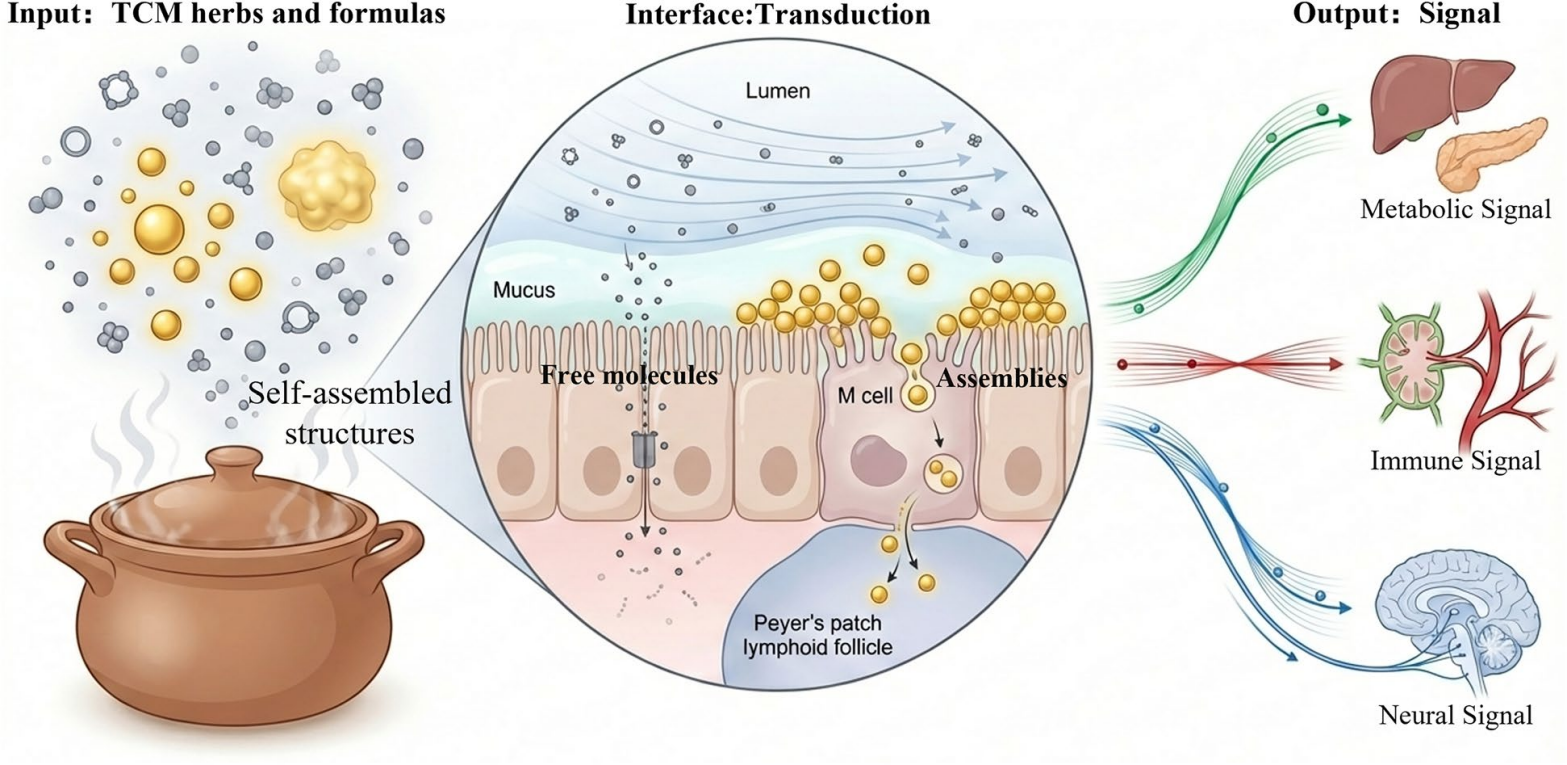
Self-assembled structures as interface-level mediators in indirect pharmacology. Herbal decoctions generate supramolecular assemblies that behave differently from free molecules. After oral delivery, these assemblies preferentially localize at the gut interface, where their persistence and surface interactions bias downstream signaling. The schematic summarizes three representative mediator routes: metabolic (gut–liver, bile acids and GLP-1), immune (gut–vascular and lymphoid pathways), and neural (gut–brain via vagal afferents)

Decoctions are particularly informative for considering self-assembly. They contain small molecules (alkaloids, flavonoids, saponins), macromolecules (polysaccharides, proteins), and inorganic components, all subjected to heating, shifting pH, changing ionic strength, and repeated concentration/dilution during preparation. These conditions encourage noncovalent association [58] and phase-like [27] behavior; therefore, the final preparation need not resemble dispersed molecular solution. From the perspective of indirect pharmacology, the key issue is not only whether nanostructures or aggregates exist, but whether their physicochemical properties alter biologically relevant exposure. Current evidence suggests that supramolecular organization can affect where constituents are concentrated, how long they are retained at interfaces such as mucus or immune niches, and which mediators become most available for local or systemic signaling [5, 8, 54] (Fig. 1). However, direct causal links between defined decoction-derived assemblies, mediator chains, and distal organ phenotypes remain limited and require critical evaluation.

In this review, we evaluate self-assembled structures as candidate material contributors to indirect pharmacology in TCM and natural products. We synthesize evidence from two related but often separate areas: naturally occurring aggregates in traditional decoctions and engineered assemblies constructed from isolated phytochemicals. We then organize the discussion around three questions relevant to indirect pharmacology: how supramolecular organization influences exposure, including pharmacokinetics and organ selectivity; how it modifies biological interfaces, particularly the gut microbiota, mucosal immunity, and barrier surfaces; and how local interfacial events may propagate into trans-organ outcomes. Finally, we revisit representative classical formulas as case studies and discuss methodological gaps and convergence strategies needed to connect material organization with mediator-linked pharmacological effects.

## The great divide between natural aggregates and designed assemblies

Research on self-assembled structures in TCM has largely developed along two parallel paths: studies of aggregates that arise spontaneously in traditional decoctions and studies of assemblies intentionally constructed from isolated natural products or phytochemicals. Both focus on supramolecular organization, but they generate different types of evidence. Naturally occurring aggregates preserve the chemical and processing context of decoctions, whereas designed assemblies provide greater control over composition, size, surface charge, and morphology. This distinction is useful for reviewing the field because the two approaches support different, and complementary, aspects of a material-based interpretation of indirect pharmacology.

### Naturally occurring aggregates and the “black box” of decoctions

One line of research treats self-assembly as an emergent physicochemical feature of decoction. Multiple experimental studies have reported that TCM decoctions are not fully molecular solutions but colloidal systems containing nano- or micro-scale aggregates, often within the approximate 50–500 nm range depending on the formula, preparation procedure, and analytical method [30, 34]. These aggregates arise through noncovalent co-assembly, in which small molecules (such as alkaloids and flavonoids) associate with abundant macromolecules, including polysaccharides and proteins, under conditions of heat and concentration typical of decoctions [44, 47]. Such findings establish that supramolecular organization is not merely an artificial formulation strategy but can be an intrinsic material feature of some traditional preparations.

For indirect pharmacology, the appeal of naturally occurring aggregates is straightforward. They may represent part of the material state to which patients or experimental animals are exposed after oral administration, at least before further transformation in the gastrointestinal environment [65]. Because the surfaces are chemically mixed, one particle can, in principle, present multiple classes of constituents to mucus, microbes, and epithelium at the same time. This makes them plausible candidates for altering local interfacial exposure. The difficulty is interpretability. Many studies can localize activity to an aggregate-rich fraction, but pinning that activity to a specific feature is much harder. The effect may depend on particle architecture, surface motifs, retained macromolecules, concentrated cargo, or release kinetics. Therefore, at present, “aggregate-associated activity” should be interpreted as an important observation rather than definitive proof that a specific supramolecular structure drives a defined mediator pathway [46].

### Designed assemblies and reductionist control

A second line of work takes a bottom-up approach, using isolated TCM-derived compounds to build defined nanostructures [54]. The emphasis is on mechanistic clarity: how specific intermolecular forces, such as electrostatic interactions between berberine and magnolol [71], drive assembly and how properties such as size, charge, and morphology translate into cellular responses. Compared with crude decoction aggregates, such systems allow more direct examination of how size, charge, morphology, and stability influence cellular uptake, mucosal interaction, release behavior, or immune recognition.

For indirect pharmacology, such simplified systems are attractive because they provide perturbable models. They make it possible to test whether a defined material parameter influences a biologically relevant interface, for instance, whether a particular surface charge favors uptake by M cells in Peyer’s patches [69]. However, the strength of these systems is also their limitation. Structures stable in simple buffers may reorganize [72] or disintegrate [59] in the chemically crowded gut lumen or within a decoction matrix. If structural integrity is lost in vivo, the proposed indirect signaling sequence may not occur. Therefore, evidence from designed assemblies demonstrates mechanistic possibilities, but it cannot be assumed to represent the behavior of natural decoction aggregates in vivo without contextual validation.

### Why convergence is needed

**Fig. 2:**
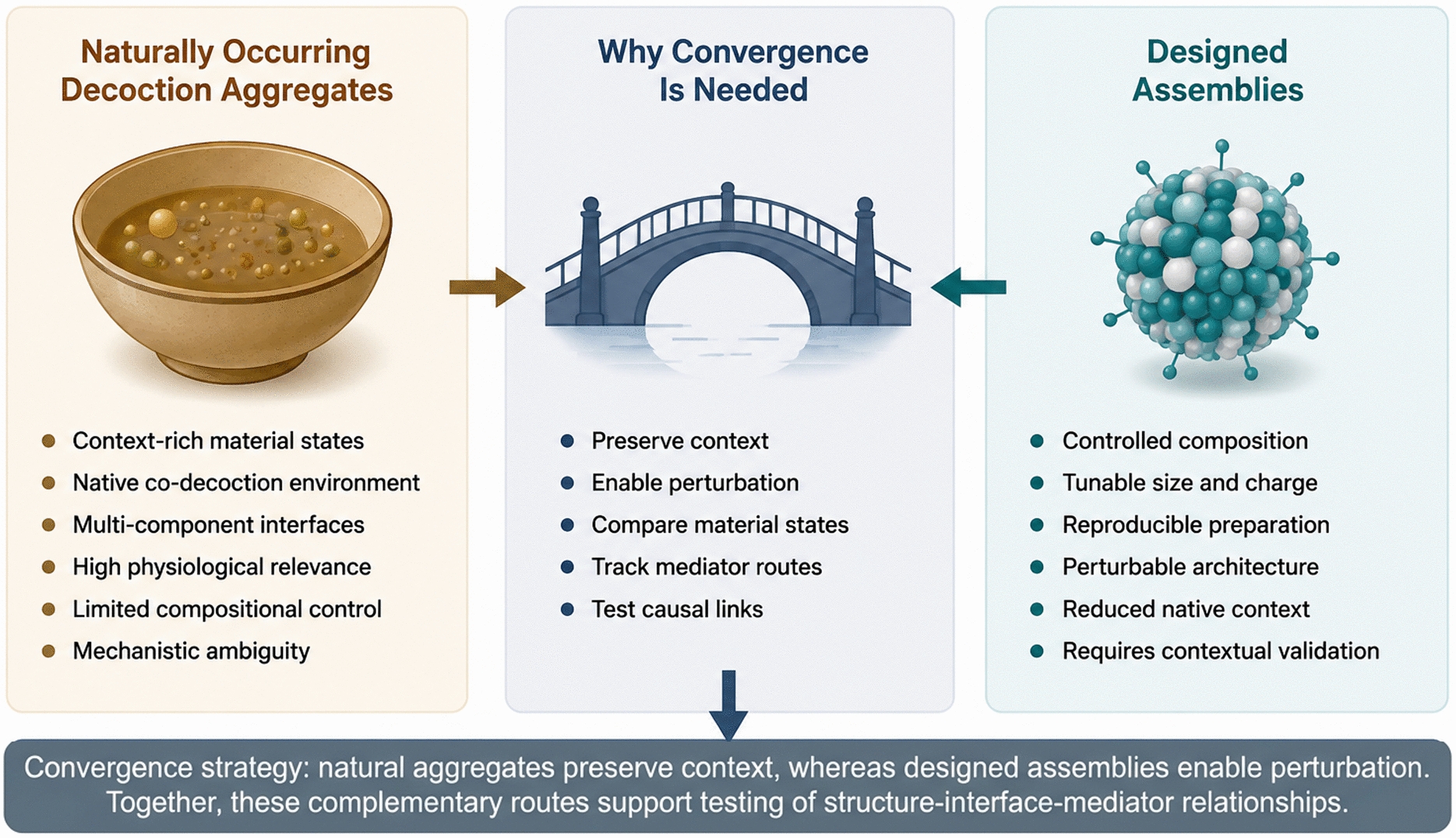
Complementary material routes for studying indirect pharmacology. Spontaneously formed decoction aggregates retain native co-decoction context, multi-component interfaces, and high physiological relevance, but have limited compositional control and mechanistic interpretability. Designed assemblies offer controlled composition, tunable physicochemical properties, reproducible preparation, and perturbable architectures, but reduce native contextual complexity and therefore require contextual validation. The figure highlights a convergence strategy in which natural aggregates preserve context and designed assemblies enable perturbation, together supporting tests of structure-interface-mediator relationships

From a systems medicine perspective, neither approach is sufficient alone. Natural aggregates preserve context but resist mechanistic dissection; engineered assemblies offer control but often lose robustness outside simplified conditions. A productive review framework should therefore treat the two approaches as complementary rather than competing. Natural aggregates can define physiologically relevant material states, while designed assemblies can serve as simplified models for testing which structural parameters are functionally important. Bridging the two approaches is essential for moving from aggregate-associated bioactivity to testable structure-interface-mediator relationships. This convergence would help clarify when material organization contributes to indirect pharmacology and when observed biological effects are better explained by co-existing soluble constituents, released cargo, or nonspecific aggregation (Fig. 2).

## From material form to systemic signal: routes of trans-organ action

Indirect pharmacology frames therapy as the correction of system-level dysregulation through inter-organ communication, not simply through single target engagement. Within this framework, self-assembled structures can be evaluated as candidate upstream material states that influence how complex preparations contact biological interfaces and generate mediator-linked responses [20, 26]. The routes discussed below should therefore be read as representative pathways supported to different degrees by current evidence, rather than as fully established mechanisms for all decoctions. We summarize the relevant assembly properties, interface-level events, mediator classes, and evidence gaps in Table 1.

**Table 1:** The influence of physicochemical properties of assemblies on biological barriers and indirect signaling routes

Physicochemical property	Value/range	Biological fate/barrier interaction	Targeted indirect route (axis)	Refs.
Particle size	< 50 nm	Rapid diffusion through mucus; potential paracellular transport	Systemic circulation (direct absorption); limited gut retention	[18, 44, 57, 69, 77]
Particle size	50–200 nm	Optimal range for uptake by M cells in Peyer’s patches; may be trapped by mucus turnover	Lymphatic system (gut–immune axis); immune training via GALT	[2, 40, 69, 73]
Particle size	> 500 nm (micro-aggregates)	Largely excluded from epithelial uptake; retained in gut lumen; may sediment on the mucosal surface	Gut microbiota modulation (gut–metabolic axis); local barrier reinforcement	[10, 29, 42, 65]
Surface charge (zeta potential)	Positive (> + 10 mV)	Strong electrostatic adhesion to negatively charged mucins (mucoadhesion)	Prolonged local residence and release; stimulation of epithelial sensors	[32, 57]
Surface charge (zeta potential)	Neutral to slightly negative	More efficient penetration through the mucus mesh (mucus-penetrating behavior); increased probability of reaching epithelial surface	Epithelial receptor interaction and/or transcytosis (context-dependent)	[18, 57, 69]
Hydrophobicity	High (lipophilic)	Partitioning into mixed micelles; incorporation into chylomicrons	Lymphatic transport (bypassing hepatic first-pass); potential targeting of adipose tissue	[12, 60]

*GALT* gut-associated lymphoid tissue. Zeta potential values are indicative and context-dependent (e.g., pH, ionic strength, and protein corona formation can alter effective surface charge and mucus interactions)

### Immune and vascular relays in trans-organ inflammation

In multi-organ diseases such as fibrosis and autoimmune disorders, immune modulation often determines systemic outcomes [23, 53]. A general principle from mucosal immunology and particulate delivery research is that particle size, surface chemistry, and persistence affect immune sampling and phagocytic recognition. These features are relevant to self-assembled structures because particulate material can resemble cues that the immune system is adapted to detect, including microbes, cell debris, and endogenous particles [2].

**Fig. 3:**
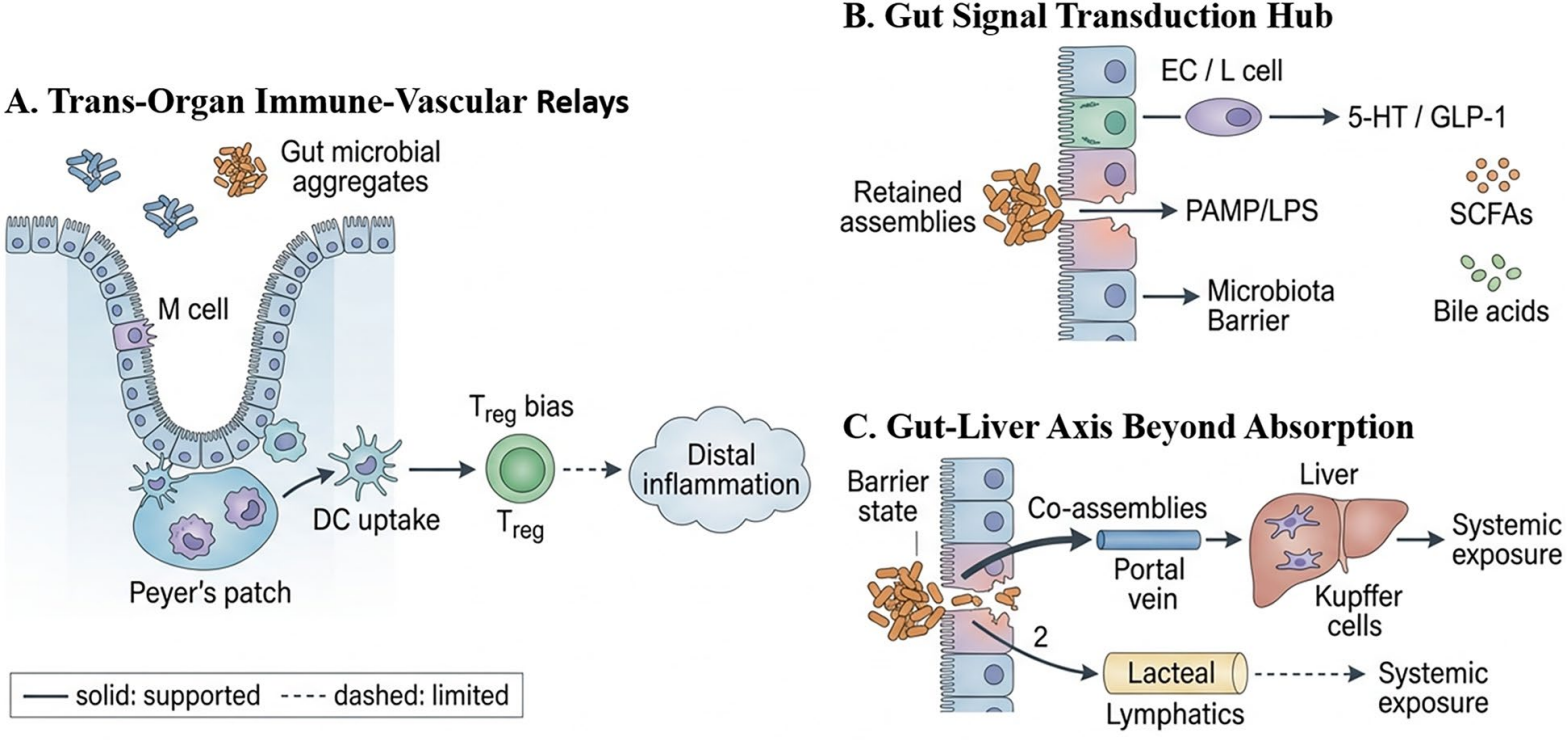
Representative trans-organ routes by which supramolecular assemblies may act within an indirect pharmacology framework. **A** Immune relay: supramolecular aggregates in the gut lumen may be sampled via M-cell-associated intestinal immune sites, with downstream immune signaling linked to distal inflammatory regulation. **B** Gut hub: assemblies retained at the mucosal surface may influence enteroendocrine signaling, barrier integrity, and microbiota-related mediator production. **C** Gut-liver routing: luminal organization and barrier status may shape portal delivery of microbial products and metabolites to the liver, while selected co-assemblies may also enter lymphatic transport

Peyer’s patches act as immune sampling sites in the gut, with microfold cells (M cells) providing a gateway for transcytosis. Nanoscale assemblies can exploit this pathway, depending on properties such as size and surface charge, to deliver materials into the gut-associated lymphoid tissue (GALT). The relevant outcome is not always systemic absorption in a pharmacokinetic sense. It can be immune “education”: dendritic cell uptake may bias responses toward tolerance or anti-inflammatory programs, including regulatory T cell (Treg)-associated responses [73]. Once programmed, immune cells and their cytokine outputs can influence inflammation at distant sites through lymphatic trafficking and circulation [14]. For TCM decoctions, this route remains best viewed as a plausible interface-to-immune relay: current evidence supports the general particle–mucosal immune principle, whereas direct proof that specific naturally occurring decoction aggregates drive defined systemic immune programs remains limited (Fig. 3A).

### The gut as a transduction hub

Within an indirect pharmacology framework, the gastrointestinal tract is more than an absorption site. It behaves as a sensing surface and major relay for systemic signaling through which luminal material, microbial activity, epithelial responses, endocrine mediators, and immune signals are integrated [38]. Self-assembled structures can engage this hub in ways that differ from freely dispersed molecules because they present as particles [67], interfaces [5], and retained depots [41] rather than as rapidly diffusing solutes. The strongest evidence at present concerns how material properties affect local exposure; the downstream connection to systemic mediator chains is more context-dependent.

Mucosal adhesion and barrier effects begin at the mucus layer, the first surface encountered by orally administered decoction. Free small molecules may diffuse and be washed out quickly, whereas supramolecular assemblies can be retained [32], especially when they incorporate polysaccharides, proteins, or carry surface charge patterns (zeta potential) that favor interaction with mucin networks [66]. Retention converts transient exposure into a local depot at the epithelial interface, extending the time window during which sensing can occur. One plausible route is via enterochromaffin (EC) cells and related enteroendocrine sensors: prolonged mechanical and chemical stimulation can promote release of serotonin (5-HT) [43] and peptides such as glucagon-like peptide-1 (GLP-1) [70]. These mediators signal through the vagus nerve and circulation, shaping satiety and metabolic homeostasis, even when the assembly itself is not distributed to the brain or pancreas. However, for most TCM-derived assemblies, direct evidence that the assembly itself, rather than released constituents or microbiota-derived metabolites, triggers EC or L-cell responses remains limited.

Barrier modulation provides a second interface-level route. Some natural product assemblies or assembly-rich fractions have been associated with improved tight-junction integrity, reduced epithelial inflammatory stress, or decreased translocation of pathogen-associated molecular patterns (PAMPs) [4, 64]. If such effects occur in vivo, they could reduce systemic low-grade inflammation by lowering luminal-to-portal inflammatory input. This mechanism is consistent with indirect pharmacology because the distal benefit would arise from improved upstream barrier function rather than from direct drug exposure in the target organ.

Microbiota-mediated effects become especially relevant when key constituents have limited oral bioavailability. Packaging into assemblies can change where these molecules end up and how long they remain intact, increasing the chance that a meaningful fraction reaches the distal gut [64]. Encapsulation/co-assembly in polysaccharide, lipid or protein matrices shields fragile phytochemicals from upper gastrointestinal early release and improves colon delivery and microbial biotransformation. Among tested colloidal carriers, sodium caseinate nanoparticles perform best for curcumin-Brassica rapa polysaccharide complex in stability, colon accumulation and gut microbiota regulation, with carrier type dominating its gastrointestinal behavior and prebiotic efficacy [19]. In the colon, assemblies can act in at least two ways. They can provide surfaces that promote bacterial adhesion and agglomeration [48] and can also behave as fermentable substrates or substrate carriers [29]. Both routes can shift the microbial metabolome, including short-chain fatty acid (SCFAs) production (e.g., butyrate and propionate) [64]. Butyrate is more than a local fuel for colonocytes; it can enter the circulation and modulate gene regulation in peripheral tissues, in part through histone deacetylase (HDAC) inhibition [16].

Another possible route involves bile acids. Assemblies that sequester primary bile acids would reduce reabsorption, push hepatic bile acid synthesis from cholesterol, and thereby influence systemic lipid profiles [28]. Direct evidence for bile acid sequestration by specific decoction-derived assemblies remains limited, and this route should be treated as a testable mechanism rather than a general property of all assemblies (Fig. 3B).

### The gut–liver axis beyond absorption

The liver is a major downstream recipient of gut-derived signals, largely via the portal circulation but also through immune and lymphatic routes. Self-assembled structures therefore raise a material-based question that is not fully captured by standard “absorption-to-plasma” pharmacokinetics: can changes in luminal organization and mucosal exposure alter the portal mixture of metabolites, microbial products, and inflammatory signals that reaches the liver?

Portal signaling links gut conditions directly to the liver. In metabolic and inflammatory disease models, increased intestinal permeability can promote translocation of LPS and related bacterial products, contributing to hepatic inflammation [42]. Assembly-mediated improvements in tight-junction integrity, microbial ecology, or luminal adsorption of pro-inflammatory factors could reduce this portal inflammatory load [45, 63]. A downstream readout may include reduced Kupffer cell activation or a shift toward less inflammatory macrophage states [10]. However, the causal sequence from a defined decoction aggregate to barrier improvement, reduced portal LPS, and altered hepatic immune tone has rarely been demonstrated in a single experimental design. This remains an important evidence gap for the gut–liver axis.

A second route involves intestinal lymphatic transport. For certain lipid-like, amphiphilic, or nanoscale co-assemblies, size and lipophilicity may favor association with intestinal lacteals rather than direct entry into blood capillaries, analogous to in part to dietary lipid transport via chylomicrons [40]. After lymphatic transport to the thoracic duct, material reaches systemic circulation before extensive hepatic first-pass metabolism [12]. This changes not only how much agent is delivered, but also when and where it appears. Nevertheless, evidence for lymphatic transport remains assembly-specific and should not be generalized to all decoction aggregates. For gut–lung or other distal axes, the more conservative interpretation is that lymphatic transport provides one possible route by which material form may influence extrahepatic exposure and immune communication [52] (Fig. 3C).

Solid arrows indicate routes supported by broader experimental evidence, whereas dashed arrows indicate assembly-specific, context-dependent, or still-testable pathways. This figure summarizes representative rather than universal mechanisms; evidence strength varies across pathways and remains strongest for interface-level events.

### A dialectical view on toxicity and anti-targets

Self-assembly should not be treated as inherently beneficial. In a systems context, “more structure” can just as easily become the wrong structure as the right one, depending on composition, stability, size, surface chemistry, and biological context. Uncontrolled aggregation in vivo, for example, precipitation triggered by abrupt pH changes in the stomach, may yield particles that are pharmacologically inert yet mechanically irritating at the mucosal surface, which would be consistent with gastrointestinal discomfort in sensitive settings.

Additional safety concern arises if assemblies or aggregate-derived particles cross into the bloodstream. There, adsorption of abundant plasma proteins can reshape the surface (a nonspecific corona), with possible downstream consequences such as complement engagement or accelerated clearance by the reticuloendothelial system (RES). These outcomes can create safety liabilities, including immune activation or off-target organ exposure, that would not be evident from efficacy endpoints alone. Therefore, defining the boundary between therapeutic assembly and pathological aggregation is part of mechanism building, not an afterthought.

## Case studies: reading TCM classics through an indirect pharmacology lens

The following examples illustrate how existing pharmacological, microbiome-related, and physicochemical findings for representative TCM formulas can be re-examined through an indirect pharmacology lens. They are not intended to prove that self-assembly is the dominant mechanism in each formula. Instead, they highlight where material organization is supported by current evidence, where it may help connect local exposure to mediator-linked systemic effects, and where causal links remain unresolved. In each case, the key question is whether assembly-associated material states add explanatory value beyond the actions of freely dissolved constituents or released cargo.

### Gegen Qinlian Decoction (GQD) and the gut-brain-metabolic axis

GQD is widely used for gastrointestinal disorders and is also discussed in the context of type 2 diabetes. A common explanation emphasizes synergy between berberine (*Coptis*) and baicalin (*Scutellaria*), alongside the long-noted issue of berberine’s low oral bioavailability [37]. From an indirect pharmacology perspective, low systemic exposure does not necessarily imply pharmacological irrelevance; it may indicate that gut-localized exposure and mediator-linked effects are important.

Several studies suggest that berberine and baicalin can co-assemble into supramolecular nanoparticles, supported by electrostatic interactions and π-π stacking [24, 80]. Separately, GQD- or berberine-related studies have linked treatment effects to changes in gut microbiota composition, short-chain fatty acids (SCFAs), including butyrate, and GLP-1-associated metabolic regulation [62]. These two evidence lines are complementary but should not be conflated. At present, the specific causal chain in which berberine–baicalin co-assembly promotes distal intestinal positioning, increases butyrate production, enhances L-cell GLP-1 secretion, and improves systemic glucose metabolism has not been fully demonstrated in a single experimental framework.

**Fig. 4:**
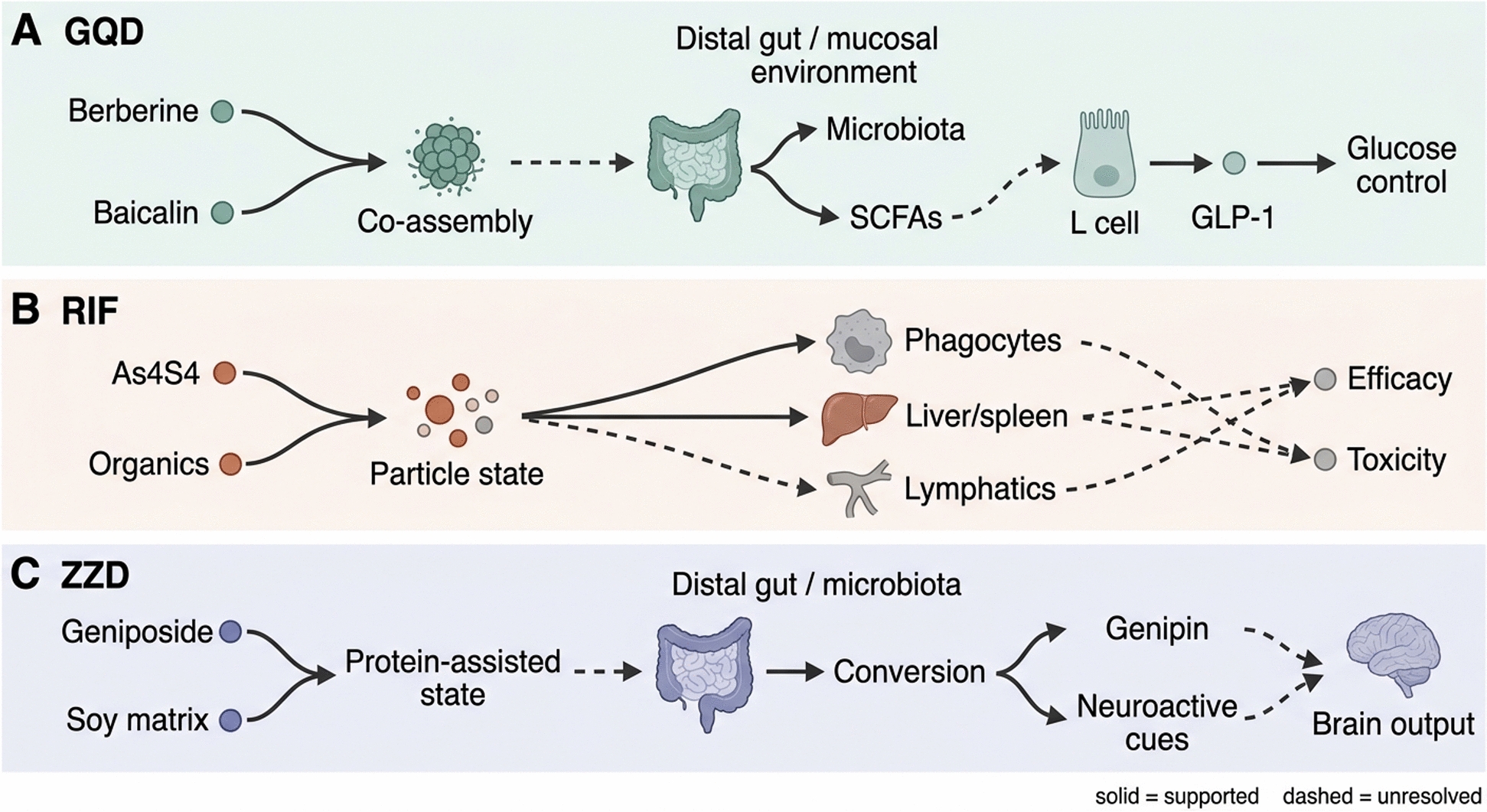
Evidence-aware schematic case studies illustrating how material organization may contribute to indirect pharmacology in representative TCM formulas. **A** Gegen Qinlian Decoction (GQD): berberine and baicalin may form co-assemblies that influence gut exposure and microbiota-related metabolic signaling, but the full co-assembly-SCFA-GLP-1-glucose control pathway remains unconfirmed. **B** Realgar-Indigo Formula (RIF): arsenic-associated material states may be modified by coexisting organics, altering particle handling and tissue distribution; this panel emphasizes a possible exposure-modifying mechanism. **C** Zhizichi Decoction (ZZD): protein-assisted organization of geniposide may affect distal gut delivery and microbial conversion, with possible downstream gut-brain signaling effects

A more conservative interpretation is therefore that GQD provides a useful case in which material organization may help explain how poorly absorbed constituents produce gut-mediated metabolic effects. The assembly-related hypothesis is most relevant to questions of intestinal localization, retention, and microbial exposure, whereas the SCFA–GLP-1 axis provides a plausible mediator route for systemic metabolic outcomes. Key unresolved issues include whether assembly-associated berberine/baicalin reaches distal gut segments in vivo, whether disruption of co-assembly alters microbiota and SCFA outputs, and whether active GLP-1 and glucose tolerance readouts, such as oral glucose tolerance test (OGTT) responses, depend on the assembled material state rather than on soluble constituents alone (Fig. 4A).

### Realgar-Indigo Formula (RIF) and the immune-vascular axis

RIF is a well-known traditional formula used in acute promyelocytic leukemia (APL) and combines realgar (As_4_S_4_), indigo naturalis, and *Salvia miltiorrhiza*. Arsenic-containing medicines illustrate an important material pharmacology problem: therapeutic activity and toxicity are both strongly influenced by chemical form, dissolution, distribution, and tissue handling. Therefore, RIF is an appropriate example for discussing how particulate or co-assembled states may alter exposure rather than simply increase or decrease total arsenic content. Reported work suggests that arsenic-containing particles may interact with coexisting organic constituents, including indirubin- and tanshinone-related compounds, thereby changing particle surface properties, dissolution behavior, or biological handling after oral administration [77].

This material perspective is relevant because particulate arsenic-associated fractions may be processed differently from rapidly dissolving arsenic species. Potential handling routes include phagocytic uptake, hepatic and splenic clearance, and possibly lymphatic or immune-cell-associated transport [21]. However, the extent to which these routes operate in vivo for RIF remains formulation- and particle-state dependent.

Thus, the self-assembly interpretation of RIF should be framed as an exposure-modifying hypothesis supported by material-state observations, rather than as a fully established explanation of efficacy and safety. This also offers a physical reading of Jun–Chen–Zuo–Shi: components assigned as “assistants” can be interpreted as modifiers of assembly and surface presentation, thereby tuning biodistribution and the balance between efficacy and toxicity [35] (Fig. 4B). The key evidence gap is whether specific dissolved versus particulate arsenic-associated fractions correlate with therapeutic differentiation signatures in hematopoietic cells while reducing hepatotoxicity markers and pathological liver accumulation. Such evidence would be needed before concluding that supramolecular organization improves the efficacy–toxicity balance of RIF.

### Aconite-licorice pairs and the gut-heart axis

Aconite is central to traditional formulas for restoring circulatory collapse, but its clinical application is constrained by the fatal cardiotoxicity of diester-diterpenoid alkaloids like aconitine. Traditionally, Aconite is paired with licorice to mitigate this risk. From a supramolecular perspective, this classic compatibility is a self-assembly phenomenon. Glycyrrhizic acid (GA), an amphiphilic saponin from licorice, spontaneously forms nanoscale micelles during decoction, physically sequestering the toxic Aconite alkaloids within their cores [15].

This assembly-related interpretation is plausible. First, this entrapment prevents rapid, massive absorption in the stomach and proximal intestine. This blunts the sharp systemic circulation peak (C_max_) responsible for acute cardiotoxicity, effectively smoothing the exposure curve [36]. Second, supramolecular sequestration acts as a spatial delay mechanism, routing a larger fraction to the distal gut. There, the intestinal microbiota acts as a bioreactor, hydrolyzing highly toxic diesters into safer derivatives before systemic entry [25]. Additionally, the GA-rich surfaces can interact with gut-associated lymphoid tissue, exerting mucosal anti-inflammatory effects that indirectly protect the cardiac endothelium via a gut-heart axis [11]. These processes provide a possible gut-mediated route by which compatibility reduces acute cardiotoxic risk.

However, the strength of this interpretation depends on evidence that the GA–alkaloid assembled state survives relevant gastrointestinal environments and changes pharmacokinetic or toxicodynamic outcomes. It should not be stated that the formula’s safety “relies entirely” on supramolecular carrier integrity. A more balanced view is that self-assembly may contribute to toxicity mitigation together with other known factors, including processing, hydrolysis, altered dissolution, metabolism, and dose control. The most important evidence gaps are whether disrupting GA-associated assemblies increases free aconitine exposure, restores early Cmax-driven cardiotoxicity, or reduces microbiota-derived detoxification products. Until such causal tests are available, GA-mediated assembly should be considered a candidate mechanism of Aconite–licorice compatibility, not the sole explanation.

### Ma Xing Shi Gan decoction and the lung-gut axis

Ma Xing Shi Gan decoction, which combines Ephedra (Mahuang) with gypsum (Shigao), is traditionally used for febrile respiratory illness. Materially, it is also an organic–inorganic mixture, which opens a different mechanistic angle [78]. Such mineral–organic associations could influence aggregation, dissolution, or intestinal exposure of Ephedra alkaloids, but direct evidence for defined calcium-mediated micro-aggregates in this formula remains limited.

Two possible indirect pharmacology routes should be considered separately. The first is a kinetic route. As gypsum dissolves, released Ca^2^⁺ may mediate associations between polysaccharides and other anionic components, as exemplified by Ca^2^⁺-induced egg-box junctions in alginate composed of repeating Ca^2^⁺-linked G-block and M-block pairs [22]. In gypsum pre-decoctions, increased inorganic dissolution and stronger functional-group interactions with Ephedra-derived constituents further suggest that Ca^2^⁺ may participate in complexation or co-association with ephedrine-type alkaloids [76]. Such calcium-mediated associations could yield micro-aggregates that change the time course of ephedra alkaloid exposure after ingestion, potentially smoothing peak concentrations linked to palpitations in susceptible individuals [62]. The second is a gut–lung immune route. Calcium-rich luminal environments or mineral–organic interfaces may influence microbial metabolism, and microbial metabolites such as desaminotyrosine (DAT) have been discussed as candidates capable of modulating antiviral immune tone [58]. Furthermore, supplementation with different calcium salts, including calcium phosphate and calcium citrate, has been shown to markedly alters gut microbial β-diversity and short-chain fatty acid (SCFA) profiles, underscoring the capacity of calcium-enriched milieus to regulate microbial metabolic activity [79]. Downstream pulmonary antiviral responses can be assessed by interferon-stimulated gene (ISG) programs and interferon (IFN)-related signaling. However, the connection between Ma Xing Shi Gan-derived assemblies, DAT-like metabolites, and lung immune protection remains speculative at present.

Therefore, this case is best presented as a more exploratory example. It is useful because it shows how inorganic components may participate in decoction material organization, but the kinetic and gut–lung immune hypotheses should not be collapsed into a single uninterrupted mechanism. Current evidence supports the plausibility of mineral–organic interactions and gut–lung immune communication in general; direct formula-specific causal evidence remains to be established.

### Zhizichi decoction and the gut-brain axis (psychological-somatic interaction)

Zhizichi decoction pairs *Gardenia jasminoides* (Zhi Zi) with Sojae Semen Praeparatum (fermented soybean) and is traditionally used for “vexation” (irritability) and insomnia-like symptoms. A material-state interpretation of Zhizichi Decoction should therefore be framed cautiously around the fermented soybean matrix. Although direct characterization of supramolecular assemblies formed specifically within Zhizichi Decoction remains limited, a recent comparable study demonstrated that geniposide forms complexes with heat-treated soybean protein isolate through hydrogen bonding involving C–N, C = O, and N–H groups, thereby altering protein secondary structure, improving geniposide stability, and increasing oral bioavailability (Lin et al.). This supports the plausibility that soybean-derived proteins or peptides in the decoction may transiently associate with gardenia-derived geniposide through noncovalent interactions, although this remains to be verified directly in Zhizichi Decoction.

Two indirect consequences are plausible but require careful separation. First, association with the protein matrix could buffer geniposide against acid-driven hydrolysis in the stomach and delay conversion to genipin, a species sometimes linked to irritation. Second, the same matrix may help deliver glycosides to the distal gut, where microbial beta-glucosidases can convert geniposide to genipin in situ [74]. In this interpretation, the key variable is not simply how much geniposide is ingested, but where and by whom it is converted. Fermentation residues can act in a prebiotic manner and shift microbial metabolism, including pathways that affect tryptophan-derived signals and γ-aminobutyric acid (GABA)-related metabolites [75]. These gut-derived cues can engage vagal and immune-to-neural routes, offering a way to influence central states without requiring direct blood–brain barrier penetration [82] (Fig. 4C).

Nevertheless, direct evidence linking soybean-assisted geniposide organization to specific gut microbial transformations and behavioral or sleep-related outcomes remains limited. Thus, Zhizichi Decoction should be presented as a gut–brain case in which material organization may modulate the site and timing of biotransformation, while the downstream neuroimmune or neuroactive metabolite pathway remains an interpretive framework requiring further validation.

Table 2 summarizes these representative self-assembled systems and the indirect signaling routes they are proposed to engage.

**Table 2:** Representative TCM self-assembled systems and their potential indirect pharmacological mechanisms

Herb/Formula	Key assemblies	Structural features	Target interface	Systemic outcome (axis)	Refs.
Gegen Qinlian Decoction (GQD)	Berberine (alkaloid) + Baicalin (flavonoid)	Self-assembled particles, electrostatic interactions and π–π stacking	Colonic lumen and mucus layer	Improved insulin sensitivity; reduced systemic inflammation (Gut–Metabolic axis)	[37, 71]
Realgar–Indigo formula (RIF)	Realgar nanoparticles (As₄S₄) + Tanshinones/Indirubin	Inorganic–organic hybrid aggregates	Peyer’s patches; reticuloendothelial system (RES)	Leukemia remission with reduced hepatic toxicity (Immune–Vascular axis)	[59]
Aconite-licorice pairs	Glycyrrhizic acid (GA) + Aconite alkaloids	Nanoscale micelles; supramolecular sequestration/physical entrapment	Proximal intestine; distal gut microbiota; GALT	Mitigation of acute cardiotoxicity; protection of cardiac endothelium (Gut-Heart axis)	[32, 64]
Ma Xing Shi Gan Decoction	Calcium ions (from Gypsum) + Ephedrine + Polysaccharides	Ca^2^⁺-bridged micro-aggregates; cross-linked networks	Gut microbiota; intestinal immune cells	Antipyretic effects; lung immune priming (Gut–Lung axis)	[30, 78]
Zhizichi Decoction (ZZCD)	Putative geniposide–soybean matrix complexes; genipin-derived pigments	Hydrogen-bonded protein–glycoside complexes; supramolecular pigment nanoparticles	Distal gut microbiota; gut–brain signaling interface	Alleviation of insomnia and anxiety (Gut–Brain axis)	[33, 74, 75, 82]

### Methodological gaps and convergence strategies

**Fig. 5:**
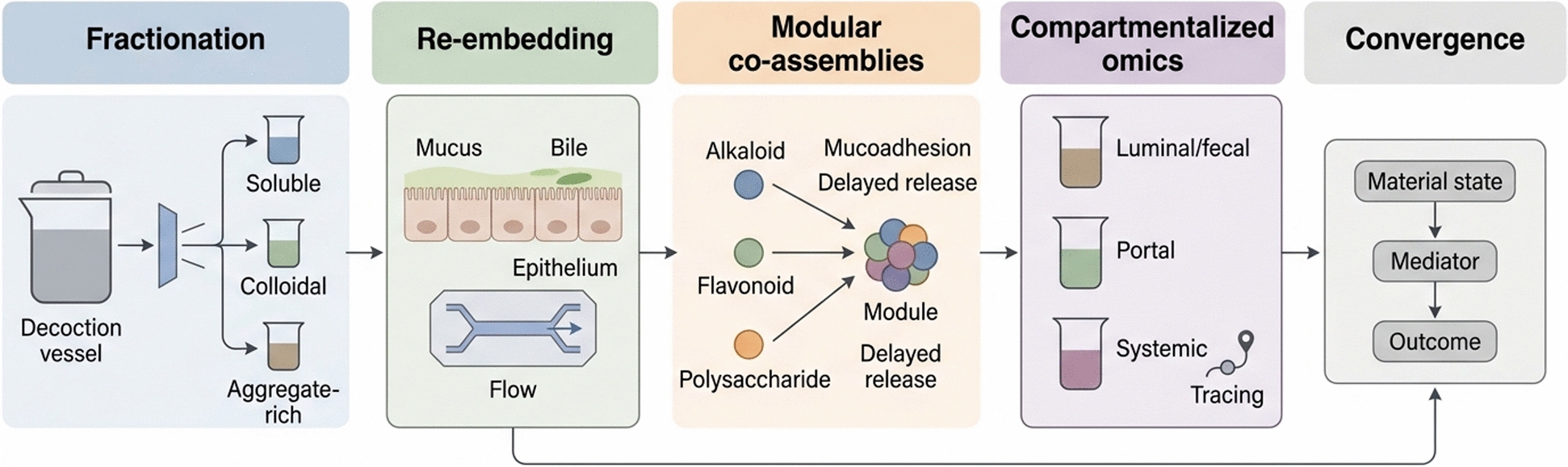
Methodological gaps and convergence strategies for linking material state, mediators, and pharmacological outcomes. Fractionation, re-embedding, modular co-assemblies, and compartmentalized omics are presented as complementary strategies whose evidence converges on relating material state to mediator-linked pharmacological outcomes, rather than as a strictly stepwise pipeline

The case studies above reveal a recurring limitation in the current literature: material characterization, mediator measurements, and systemic pharmacological outcomes are often examined separately. As a result, it remains difficult to determine whether a self-assembled state is causally involved in an indirect pharmacological effect or is merely associated with active constituents. Descriptive characterization of particles or aggregates is therefore necessary but not sufficient. Below, we summarize several methodological gaps and convergence strategies that may help connect material-state characterization with mediator-linked pharmacological outcomes. These strategies should be viewed as tools for evaluating specific structure–interface–mediator relationships rather than as a universal research program for all decoctions (Fig. 5).

### Bottom-up fractionation of decoction assemblies

A first methodological gap concerns the identification of functionally relevant material populations within crude decoctions. In many decoctions, the particulate fraction is treated as inert sediment and discarded, yet it is often the most assembly-rich component [65]. Operational fractionation methods, such as differential centrifugation, ultrafiltration, size-exclusion separation, or field-flow fractionation, can separate decoctions into soluble, colloidal, and aggregate-enriched fractions for parallel structural and biological analysis [18].

The value of this approach is that it links biological readouts to defined material populations rather than to the crude decoction as a whole. Downstream screening should reflect the proposed indirect route. For example, fractions relevant to gut–metabolic signaling could be tested for GLP-1 secretion in enteroendocrine cell models such as STC-1 cells, whereas immune-related fractions could be evaluated using macrophage or dendritic-cell co-culture systems and cytokine readouts [55]. Fractionation alone cannot prove causality, but it can identify which material populations carry mediator-linked activity and should be prioritized for mechanistic perturbation, structural disruption, or in vivo validation [31].

### Contextual re-embedding: gut-relevant matrices and microfluidic models

A second methodological gap is that assemblies that appear well defined in buffer often reorganize once they encounter bile salts, mucus, dietary colloids, or the chemical background of the parent decoction [1]. Contextual re-embedding therefore refers to deliberately re-challenging assemblies with gut-relevant matrices or simulated physiological fluids and examining which supramolecular states persist [60]. For indirect pharmacology, these transitions should not be dismissed as experimental noise; they may determine where mediators are generated and which biological interfaces are exposed.

Microfluidic platforms provide a practical way to do this while retaining spatial resolution [39]. Gut-on-a-chip systems, optionally coupled to downstream liver modules, allow assemblies to be introduced luminally while epithelial responses, mediator release, and metabolic readouts are sampled under flow and mucus-like conditions [56]. These models are reductionist and do not replace animal or clinical validation. Their main value is to test whether a proposed structure–mediator link persists under more physiologically relevant interface conditions.

### Mesoscale modular co-assemblies

A third methodological gap lies between overly complex crude decoctions and overly simplified single-compound systems. Whole decoctions preserve context but are difficult to perturb mechanistically, whereas isolated compounds often lose the emergent behavior produced by multicomponent interactions. A practical middle ground is to work with mesoscale modular co-assemblies: small, recurring sets of constituents that are sufficient to recreate a characteristic assembly motif, such as an alkaloid–flavonoid–polysaccharide cluster. These modules retain emergent properties like mucoadhesion or delayed release, while remaining amenable to reproducible preparation [49, 51].

These modules can be useful because they retain selected emergent properties, such as mucoadhesion, delayed release, cargo protection, or surface-mediated biological interaction, while remaining experimentally reproducible. However, they should be treated as models of selected interaction motifs, not as substitutes for the complete formula. Structural methods, including small-angle X-ray scattering (SAXS), cryogenic electron microscopy (cryo-EM), dynamic light scattering, zeta-potential analysis, rheology, and mucin-binding assays, can be combined to relate internal organization and surface properties to interface-level behavior [9, 50]. This approach can help identify which material parameters are functionally relevant, but any conclusions require validation against the original decoction or biologically relevant matrices.

### Compartmentalized omics and flux analysis

A fourth methodological gap concerns sampling location. When pharmacological effects are mediated indirectly, systemic plasma is often the wrong place to start. Many relevant signals are generated in the lumen or at the mucosa, relayed through portal circulation, and only later reflected in peripheral blood. A compartmentalized sampling strategyspanning luminal content (or fecal water), portal vein blood, and systemic plasmahelps make this sequence explicit [6, 81].

Stable-isotope tracing and flux analysis can further distinguish direct exposure models from mediator-driven indirect models. By following labeled constituents or inputs, it becomes possible to ask whether intact compounds reach circulation at levels compatible with a direct exposure model, or whether efficacy instead tracks with delayed changes in host- or microbiota-derived mediators such as secondary bile acids [17, 68]. These methods do not establish causality on their own, but they can narrow competing explanations and identify which links require perturbation-based validation.

## Conclusions and perspectives

Indirect pharmacology shifts attention away from single-target “magic bullets” toward therapeutic regulation distributed across biological interfaces, mediator networks, and inter-organ communication. Within this framework, self-assembled structures in TCM decoctions can be considered candidate material contributors that influence how complex preparations localize, persist, and interact with gut microbiota, mucosal immunity, epithelial barriers, and related interfaces. Current evidence supports the view that such material states can alter exposure and interface behavior, although direct causal links to defined mediator chains and distal organ phenotypes remain limited. Accordingly, self-assembly provides a plausible framework for understanding how local interfacial events may contribute to measurable multi-organ outcomes, rather than a universally established explanation for them. The next step is to make the structure–mediator–function relationship testable rather than assumed. Demonstrating that an assembly exists is only the starting point; the stronger claim is that maintaining (or disrupting) its integrity predictably alters a defined mediator chain and downstream inter-organ readouts. Approaches that combine supramolecular characterization with compartment-aware physiology and causal perturbations should help clarify when “holistic” effects reflect organization and interface biology, rather than simply co-occurrence of many constituents. A major unresolved issue is how to test structure–mediator–function relationships directly rather than infer them from co-occurring observations. Demonstrating that an assembly exists is only the starting point; stronger evidence would require showing that preserving, disrupting, or reconstituting a defined material state changes mediator profiles and distal readouts in a reproducible and context-consistent manner. Progress in this area will depend on integrating supramolecular characterization, context-relevant biological models, compartment-aware sampling, and perturbation-based validation. If these links can be clarified, the language of “holistic regulation” may be translated into experimentally testable, material-informed mechanisms relevant to systems pharmacology.

## Data Availability

No datasets were generated or analysed during the current study.

## Abbreviations

APL: Acute promyelocytic leukemia
C_max_: Maximum plasma concentration
DAT: Desaminotyrosine
EC cells: Enterochromaffin cells
GABA: γ-Aminobutyric acid
GA: Glycyrrhizic acid
GALT: Gut-associated lymphoid tissue
GLP-1: Glucagon-like peptide-1
GQD: Gegen Qinlian Decoction
HDAC: Histone deacetylase
IFN: Interferon
ISG: Interferon-stimulated gene
LPS: Lipopolysaccharide
M cells: Microfold cells
OGTT: Oral glucose tolerance test
PAMPs: Pathogen-associated molecular patterns
RES: Reticuloendothelial system
RIF: Realgar-Indigo Formula
SAXS: Small-angle X-ray scattering
SCFAs: Short-chain fatty acids
TCM: Traditional Chinese Medicine
Treg: Regulatory T cell
5-HT: 5-Hydroxytryptamine/serotonin
ZZCD: Zhizichi Decoction
